# Comparison of the Detection and Ligation of Patent Processus Vaginalis Between Laparoscopy-Assisted Transscrotal Orchiopexy and Single Scrotal Incision Orchiopexy

**DOI:** 10.3389/fsurg.2021.819057

**Published:** 2022-01-31

**Authors:** Weiguang Zhao, Cheng Su, Shoulin Li, Zengnan Mo

**Affiliations:** ^1^Guangxi Key Laboratory for Genomic and Personalized Medicine, Guangxi Medical University, Nanning, China; ^2^Department of Urology, The First Affiliated Hospital of Guangxi Medical University, Guangxi Medical University, Nanning, China; ^3^Center for Genomic and Personalized Medicine, Guangxi Medical University, Nanning, China; ^4^Department of Pediatric Urology, Shenzhen Children's Hospital, Shenzhen, China; ^5^Guangxi Collaborative Innovation Center for Genomic and Personalized Medicine, Guangxi Medical University, Nanning, China

**Keywords:** cryptorchidism, processus vaginalis, laparoscopy, orchiopexy, undescended testes

## Abstract

This study aimed to compare the detection and ligation of patent processus vaginalis (PPV) between laparoscopy-assisted transscrotal orchidopexy (LATO) and single scrotal incision orchiopexy (SSIO) for low palpable undescended testis (UDT). We performed a retrospective medical record review of transscrotal orchidopexies performed for low palpable UDT at our institution from 2017 to 2019; 33 and 39 boys underwent LATO and SSIO, respectively. Data collection included patient demographics, incidence of PPV, operative time, and clinical outcomes. All 95 testes were delivered into the scrotum. There was no significant difference between the two groups with respect to patients' age, side, and mean operative time. The incidence of PPV in the LATO group was significantly higher than that in the SSIO group (56.52 vs. 34.69%, *P* = 0.04). The incidence of contralateral PPV in the LATO group was 45%. One patient in the SSIO group underwent unilateral PV ligation and laparoscopic exploration revealed bilateral PPV owing to metachronous contralateral hydrocele. One patient in the LATO group demonstrated obliterated PV in the initial transscrotal procedure, but an ipsilateral PPV was found in the latter laparoscopic procedure. In conclusion, LATO has a higher detection rate and higher ligation of the PPV than SSIO, suggesting that, LATO may help reduce recurrent PPV-related issues. However, long-term follow-up results are needed to evaluate the advantages and disadvantages in a larger case series.

## Introduction

Processus vaginalis (PV) represents an outpouching of the parietal peritoneum, which is necessary for the descent of the testes and obliterates after testicular migration is completed (1). Non-obliteration of the PV results in inguinal hernia, hydrocele, and undescended testis (UDT) (2). The identification and high ligation of the patent PV (PPV) is one of the principal steps in inguinal and transscrotal orchidopexies.

Traditional transscrotal orchidopexy is accomplished by a single transscrotal incision and is mainly advocated for low-positioned UDT, which could be manipulated at least to the entrance of the scrotum. Despite the promising results of transscrotal orchiopexy reported in the literature, it has been argued that the technical complexity and inability to perform a high ligation of the PV through the single scrotal incision prevent this technique from gaining widespread acceptance (3–7). In addition, intraoperative findings suggested that the PV is not wide open in most UDT and is too small to be identified. Meanwhile, it is not possible to accurately define the proximal extent of the cord in all cases (8). Therefore, the sensitivity of detection of PPV by single scrotal incision orchiopexy (SSIO) is obscure.

In recent years, laparoscopic percutaneous extraperitoneal closure has been considered a routine treatment for hydrocele and inguinal hernia in many medical centers (9–12). Compared with traditional inguinal repair, the processus, if patent, is dilated via pneumoperitoneum, and magnifying the area using laparoscopy makes it easier to distinguish the orientation of the processus (10). In 2018, Ma et al. (13) first introduced laparoscopy-assisted transscrotal orchidopexy (LATO), which was laparoscopic percutaneous extraperitoneal closure combined with transscrotal orchidopexy, to manage palpable inguinal UDT. Similarly, in 2020, Saka et al. (14) reported a similar technique for treating intracanalicular and extracanalicular UDT with promising results. Both studies reported a patent rate of ipsilateral PV as high as nearly 100%.

Although the LATO technique has been reported, further evaluation of this new technique is needed. We hypothesized that compared to SSIO, LATO might represent an improvement in distinguishing and ligating PPV. However, for transscrotal orchiopexy in patients with low palpable UDT, no report has been published to support this assumption. Thus, the current study retrospectively investigated and compared the management of PPV between LATO and SSIO in patients with low palpable UDT.

## Materials and Methods

### Patients

This single-centered retrospective study reviewed the charts of 72 patients who underwent transscrotal orchiopexy for low palpable UDT by a single surgeon from November 2017 to September 2019 with a minimum of 12-months follow-up. A low palpable UDT was defined as a testis that could be manipulated to at least the entrance of the scrotum. All children were examined on at least two occasions, including preoperatively and again under general anesthesia, and the ultimate surgical approach decision was made during surgery. Exclusion criteria included retractable testis, ectopic testis, non-palpable testis, previous groin operation, and lack of operative data records.

The study cohort was divided into two groups based on the surgery performed according to the guardians' or patients' requests as follows: the SSIO group who underwent single transscrotal orchidopexy, and the LATO group who underwent laparoscopy-assisted transscrotal orchidopexy. We recorded and compared the age at surgery, side of UDT, patency of the PV, and operative time. In general, during regular postoperative follow-up, we evaluated the position and status of the testis at one, 3 months, and annually at least until they reached puberty to ensure that no complications had developed. Success was defined as mid or lower scrotal position of the testis.

### Surgical Technique for SSIO

**Figure 1:**
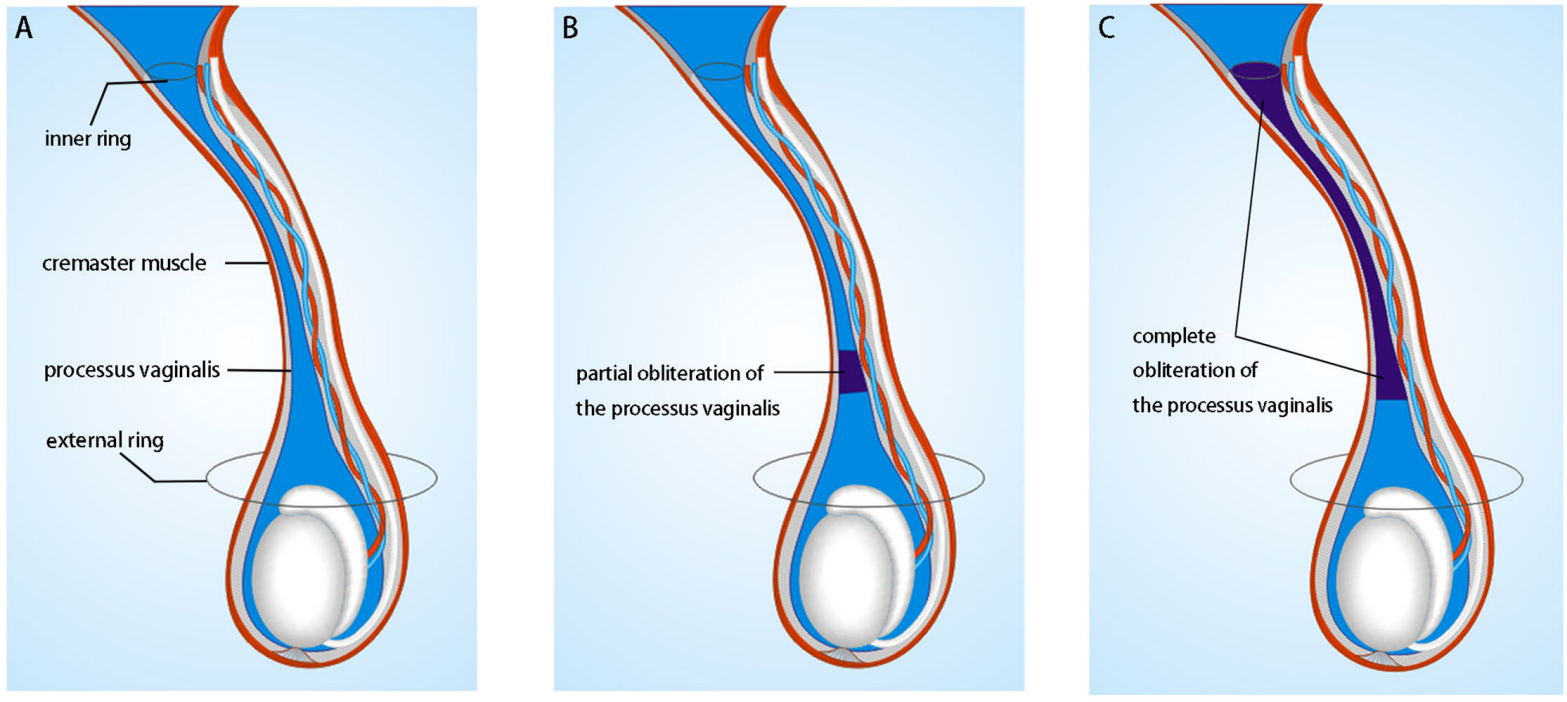
Schematic drawing showing the intraoperative findings of the processus vaginalis: **(A)** wholly open if a communication existed from the inner ring to the tunica vaginalis of the testis; **(B)** partially open when it opened at the internal ring but obliterated distally before reaching the testis; **(C)** complete obliteration from the internal ring to the upper extent of the tunica vaginalis.

A transverse incision (10–15 mm in length) was made along the middle scrotum, followed by creating an adequate dartos pouch for relocation. The testis was then exteriorized through an incision. With traction on the cut gubernaculum, the external spermatic fascia of the testis was released up to the external ring region. After blunt transection between the cremasteric fascia and the PV plane (Figure 1), the cremasteric fascia was transected. The anterior wall of the tunica vaginalis was opened longitudinally. The PV was considered wide open if a communication existed from the PV to the tunica vaginalis of the testis (Figure 1A), and partially open when it opened at the internal ring but is obliterated distally before reaching the testis (Figure 1B). PV ligation was performed as much as possible with the aid of sac traction. The PV was considered closed if no orifice along the spermatic cord was found to communicate with the abdominal cavity (Figure 1C). The testis was confirmed with the correct axial direction and standardly fixed.

### Surgical Technique for LATO

The bilateral internal inguinal ring was checked carefully under laparoscopy, and if the PPV was confirmed, we performed simultaneous enclosure. Our technique for laparoscopic percutaneous extraperitoneal closure has been previously reported (9). Briefly, a hernia needle with 2–0 non-absorbable suture was carefully introduced around the medial half-circle of the internal ring, over the vas deferens and spermatic vessels, and then pierced the peritoneum into the peritoneal cavity. After the suture was held by grasping forceps, the needle was passed around the lateral half of the internal ring, entering the peritoneum at the same location, withdrawing together with the suture so that the internal ring was encircled. The suture was tied tightly; thus, complete extraperitoneal ligation of the hernia sac was completed. The scrotal orchidopexy technique was similar to the SSIO technique described above, except that there was no need for transseptal PV ligation.

### Statistical Analysis

Continuous variables and categorical variables were compared using Student's *t*-test and Chi-square test or Fisher exact test, respectively. Statistical analyses were performed using the SPSS statistical software (version 26.0; IBM Corp, SPSS, Inc., Chicago, IL, USA).

## Results

All 95 undescended testes of 72 patients were successfully delivered to the mid or lower scrotal positions. The characteristics of all included patients are shown in Table 1. In the overall study population, there were no significant differences between patients who underwent SSIO and those who underwent LATO with respect to age, laterality, and operative time. However, patients who underwent SSIO had a significantly lower PPV rate than those who underwent LATO (34.69 vs. 56.52%, *P* = 0.04).

**Table 1:** Patient characteristics and surgical outcome by surgical procedures.

	**SSIO**	**LATO**	***p*-value**
Patients	39	33	
No. orchidopexies	49	46	
Age, year, Mean (SD)	4.21 (3.29)	3.42 (2.38)	0.25
**Side of UDT**			0.12
Right side, *n* (%)	12 (30.76%)	13 (39.39%)	
Left side, *n* (%)	17 (43.58%)	7 (21.21%)	
Bilateral, *n* (%)	10 (25.64%)	13 (39.39%)	
**Processus vaginalis**
PPV, *n* (%)	17 (34.69%)	26 (56.52%)	0.04^*^
cPPV, *n* (%)	None	9 (45%)	
**Operative time, min**			0.12
Mean (SD)	46.15 (12.75)	50.97 (13.52)	
Hemorrhage	2 patients	0	

*SSIO, single scrotal incision orchiopexy; LATO, laparoscopic assisted transscrotal orchidopexy; SD, standard deviation; UDT, undescended testis; PPV, patent processus vaginalis; cPPV, contralateral patent processus vaginalis*.

* *Statistically significant*.

In the SSIO group, one patient who underwent unilateral PV ligation developed a metachronous contralateral hydrocele after 10 months; subsequent laparoscopic exploration demonstrated bilateral PPV, and bilateral percutaneous extraperitoneal closure was performed.

In the LATO group, 32 patients underwent closure of the PPV before the transscrotal approach, whereas one patient underwent the opposite order. This patient demonstrated an obliterated PV in the initial transscrotal procedure, but a PPV was observed in the latter laparoscopic procedure.

None of the patients underwent conversion to the inguinal approach. Scrotal hematoma occurred in two early cases in the SSIO group: one of them underwent hemostasis operation, whereas the other was conservatively treated and healed at 1-month post-surgery.

**Figure 2:**
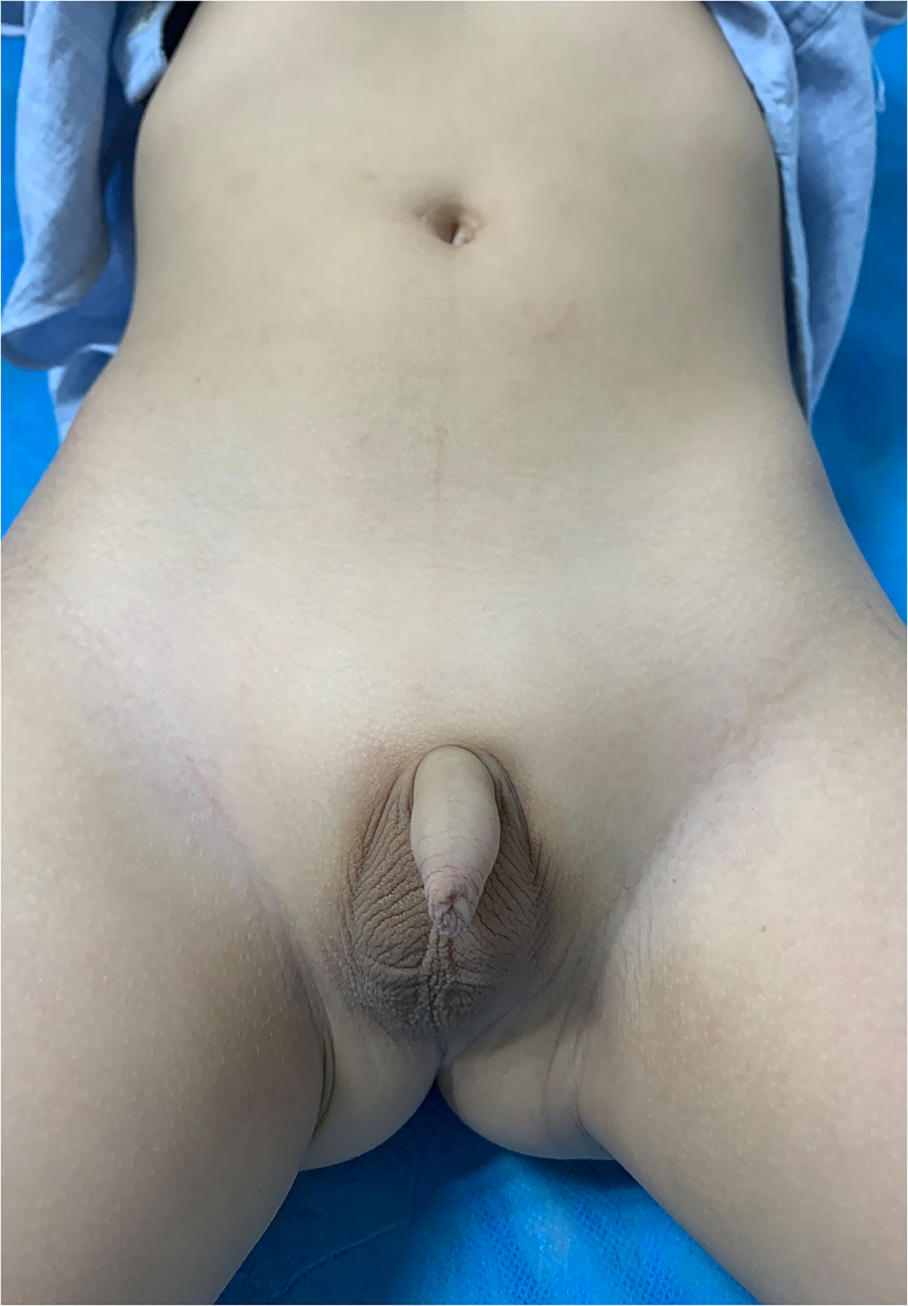
Cosmetic result of a patient with bilateral UDT underwent LATO at the 12-month follow-up.

Postoperative follow-up was feasible in all patients, with a median period of 22 months (range, 12–31 months). Scar formation was minimal (Figure 2).

## Discussion

This study supports the role of transscrotal orchiopexy as a feasible and safe strategy for management of low palpable UDT. The technique is well-tolerated and cosmetically pleasing and is associated with a short operative time, low complication rates, and favorable testicular descend. Meanwhile, in this retrospective comparison, we support some arguments for the use of SSIO in the treatment of PPV in patients with low palpable UDT as follows: shortcomings of the single transscrotal approach can be overcome by combining it with a laparoscopy-assisted approach. To the best of our knowledge, this retrospective comparison of laparoscopy-assisted and single transscrotal orchidopexy has not been previously reported.

First, SSIO cannot accurately detect the ipsilateral PPV. In the transscrotal approach, patency of the PV is determined by observing the fluid in the PV and/or probing from below, as well as by opening the anterior processus to the point of obliteration in most cases. However, these determination methods are difficult in some circumstances, especially when the PV opens in the inner ring but wholly or partially obliterates along the cord (15). According to previous studies, the detection rates of PPV mostly range from 20 to 59% in transscrotal orchiopexy cases (16–19), which are lower than the rate of PPV (90%) in laparoscopic orchiopexy for palpable inguinal UDT (20, 21) and that of PPV (100%) in LATO for low palpable UDT (13, 14). In fact, the transscrotal approach has also been used in hydrocele repair in pediatric patients, with a patency rate of 66% (20/30 cases) in the 0-to 5-year age group (22). However, the patency rate of PV can be as high as 97.7% (43/44 cases) to 99.9% (283/284 cases) in comparable age patients treated with laparoscopic percutaneous extraperitoneal closure (10, 12). Meanwhile, the high sensitivity (99.4%) and specificity (99.5%) of laparoscopy have been reported for PPV detection (23). In our LATO group, one patient had an obliterated PV in the initial transscrotal procedure, but the ipsilateral PV was confirmed patent in the latter laparoscopic procedure. Moreover, the SSIO group detected fewer PPVs than the LATO group (34.69 vs. 56.52%, *P* = 0.04). These data indicate that the laparoscopic approach has a superior sensitivity in detecting PPV than the single transscrotal approach due to advantages, such as the straightforwardness and magnified visualization.

Second, conventional transscrotal surgery cannot confirm whether cPPV exists. In our SSIO group, one patient developed metachronous contralateral hydrocele, and a second surgery was performed. However, laparoscopy can be used for the simultaneous detection of the contralateral pathology and accurate diagnosis of cPPV (9). If cPPV exists, ligation can be performed to close it and avoid a second surgery. The prevalence of cPPV was 45% (9/20 testes) in our LATO group, which is comparable to that reported by Ma (46.8%, 15/32 low palpable UDT) (13) and You (46.8%, 223/677 inguinal palpable UDT) (20).

Third, the SSIO cannot ligate PV sufficiently high to the inner ring level. Although the inguinal canal is short in children, and relatively high ligation is reported to be possible by using traction in transscrotal incision (24), continuous concerns have been reported on deficient low ligation of the PV (17, 25, 26). In fact, the incidence of metachronous ipsilateral inguinal hernias after scrotal orchidopexy with PV ligation is reportedly 3% (16). In addition, no report has adopted laparoscopy as modality to confirm whether transscrotal ligation of the PPV reached the level of the inner ring. In our SSIO group, one patient underwent unilateral PV ligation, but after 10 months, a laparoscopic exploration owing to metachronous contralateral hydrocele found that the bilateral inner ring was unclosed; therefore, a bilateral percutaneous extraperitoneal closure was performed. Although no ipsilateral complications arose from the SSIO group during the relatively short follow-up period, our data show that the ligation of the PV in the transseptal approach cannot reach as high as the level of the inner ring.

Fourth, the surgical difficulty and technical complexity of performing high ligation of the PV through a scrotal incision have been of concern (7, 17). In some cases, conversion to an inguinal approach was necessary because of the difficulty in isolating the PPV (27, 28). Because transscrotal PV ligation was not needed in LATO, our data show that the operative time was almost similar between SSIO and LATO procedures (46.15 ± 12.75 vs. 50.97 ± 13.52 min, *p* = 0.12). We suggest replacing transscrotal ligation with laparoscopic percutaneous extraperitoneal closure, which has been a routine approach for treating hydrocele and inguinal hernia in many centers.

The discrepancy in PPV detection between the two approaches can be explained not only by the different incisions, but also by the undefined obliteration process of the PV. Although the exact biological mechanism of the etiology and obliteration of the PV is not completely understood, we hypothesize that the part of the PV superior to the testis is obliterated at first, then cephalad up to the inner ring. Finally, the tunica vaginalis progressively obliterates and adheres to the testes after 50 years of age (29). This hypothesis could explain why the PV appeared to be obliterated in transscrotal surgery but was patent under laparoscopy.

A potential bias of our study is reflected in the significantly lower PPV rate (58.1%) of our LATO approach group compared with that (100%) of the published LATO approach groups (13, 14). The patency rates of PV differ according to testicular location in cryptorchidism; in particular, a distal testicular location is associated with a low patency PV rate (30). In our patient population, prescrotal UDT was more prevalent. This explains the PPV rate discrepancy observed between our LATO group and the published LATO groups.

The present study had several limitations. It is a retrospective study and is subject to all biases due to its design. Additionally, most LATO cases underwent closure of the PPV before the transscrotal approach; in case of the opposite order, more convincing results of PPV detection and ligation by the transscrotal approach would be obtained. Moreover, a prospective trial in a larger cohort matching all children groups with different surgical approaches, age, and location of the testis would further confirm these findings. In conclusion, LATO has a higher detection rate and higher ligation of PPV than SSIO. This difference is likely due to the technical advantages offered by laparoscopy and the undefined obliteration process of the PV. Further larger case series, prospective randomized, and long-term follow-up studies are needed to confirm these findings.

## Data Availability Statement

The original contributions presented in the study are included in the article/supplementary material, further inquiries can be directed to the corresponding author/s.

## Ethics Statement

The studies involving human participants were reviewed and approved by institutional Ethics Committee of Shenzhen Children's Hospital. Written informed consent to participate in this study was provided by the participants' legal guardian/next of kin. Written informed consent was obtained from the minor(s)' legal guardian/next of kin for the publication of any potentially identifiable images or data included in this article.

## Author Contributions

WZ and ZM: conception and design. SL and ZM: administrative support. WZ and SL: provision of study materials or patients, collection, and assembly of data. WZ and CS: data analysis and interpretation. All authors manuscript writing and final approval of manuscript.

## Funding

This study was supported by grants from Sanming Project of Medicine in Shenzhen, China (SZSM201612013), Shenzhen Fund for Guangdong Provincial High-level Clinical Key specialties (Grant No. SZXK035), and National Natural Science Foundation of China (Grant No. U1904208).

## Conflict of Interest

The authors declare that the research was conducted in the absence of any commercial or financial relationships that could be construed as a potential conflict of interest.

## Publisher's Note

All claims expressed in this article are solely those of the authors and do not necessarily represent those of their affiliated organizations, or those of the publisher, the editors and the reviewers. Any product that may be evaluated in this article, or claim that may be made by its manufacturer, is not guaranteed or endorsed by the publisher.
